# The Effects of SEA0400 on Ca^2+^ Transient Amplitude and Proarrhythmia Depend on the Na^+^/Ca^2+^ Exchanger Expression Level in Murine Models

**DOI:** 10.3389/fphar.2017.00649

**Published:** 2017-09-21

**Authors:** Nils Bögeholz, Jan S. Schulte, Sven Kaese, B. Klemens Bauer, Paul Pauls, Dirk G. Dechering, Gerrit Frommeyer, Joshua I. Goldhaber, Uwe Kirchhefer, Lars Eckardt, Christian Pott, Frank U. Müller

**Affiliations:** ^1^Division of Electrophysiology, Department of Cardiovascular Medicine, University Hospital Münster Münster, Germany; ^2^Institute of Pharmacology and Toxicology, University of Münster Münster, Germany; ^3^Cedars-Sinai Medical Center, Heart Institute Los Angeles, CA, United States

**Keywords:** Na^+^/Ca^2+^ exchanger, arrhythmia, heart failure, novel antiarrhythmic strategies, Ca^2+^ cycling, transgenic mice

## Abstract

**Background/Objective:** The cardiac Na^+^/Ca^2+^ exchanger (NCX) has been identified as a promising target to counter arrhythmia in previous studies investigating the benefit of NCX inhibition. However, the consequences of NCX inhibition have not been investigated in the setting of altered NCX expression and function, which is essential, since major cardiac diseases (heart failure/atrial fibrillation) exhibit NCX upregulation. Thus, we here investigated the effects of the NCX inhibitor SEA0400 on the Ca^2+^ transient amplitude and on proarrhythmia in homozygous NCX overexpressor (OE) and heterozygous NCX knockout (hetKO) mice compared to corresponding wild-types (WT_OE_/WT_hetKO_).

**Methods/Results:** Ca^2+^ transients of field-stimulated isolated ventricular cardiomyocytes were recorded with fluo-4-AM or indo-1-AM. SEA0400 (1 μM) significantly reduced NCX forward mode function in all mouse lines. SEA0400 (1 μM) significantly increased the amplitude of field-stimulated Ca^2+^ transients in WT_OE_, WT_hetKO_, and hetKO, but not in OE (% of basal; OE = 98.7 ± 5.0; WT_OE_ = 137.8 ± 5.2^*^; WT_hetKO_ = 126.3 ± 6.0^*^; hetKO = 140.6 ± 12.8^*^; ^*^p < 0.05 vs. basal). SEA0400 (1 μM) significantly reduced the number of proarrhythmic spontaneous Ca^2+^ transients (sCR) in OE, but increased it in WT_OE_, WT_hetKO_ and hetKO (sCR per cell; basal/+SEA0400; OE = 12.5/3.7; WT_OE_ = 0.2/2.4; WT_hetKO_ = 1.3/8.8; hetKO = 0.2/5.5) and induced Ca^2+^ overload with subsequent cell death in hetKO.

**Conclusion:** The effects of SEA0400 on Ca^2+^ transient amplitude and the occurrence of spontaneous Ca^2+^ transients as a proxy measure for inotropy and cellular proarrhythmia depend on the NCX expression level. The antiarrhythmic effect of SEA0400 in conditions of increased NCX expression promotes the therapeutic concept of NCX inhibition in heart failure/atrial fibrillation. Conversely, in conditions of reduced NCX expression, SEA0400 suppressed the NCX function below a critical level leading to adverse Ca^2+^ accumulation as reflected by an increase in Ca^2+^ transient amplitude, proarrhythmia and cell death. Thus, the remaining NCX function under inhibition may be a critical factor determining the inotropic and antiarrhythmic efficacy of SEA0400.

## Introduction

Approximately 25% of cardiovascular mortality has been attributed to sudden cardiac death (Priori et al., 2015). Except for betablockers, conventional antiarrhythmic drugs targeting Na^+^, Ca^2+^, or K^+^ channels have not been shown to prevent sudden cardiac death. Due to these unsatisfying results, much effort has been made to identify alternative targets to prevent from life threatening arrhythmias. In this regard, heart failure—the most prevalent precondition for sudden cardiac death—is accompanied by an upregulation of the cardiac Na^+^/Ca^2+^ exchanger (NCX) (Menick et al., 2007). NCX is the dominant mechanism for the extrusion of Ca^2+^ out of the cell. In its electrogenic forward mode NCX extrudes 1 Ca^2+^ ion in exchange for 3 Na^+^ ions thus generating a net electrical inward current that depolarizes the cellular membrane potential. In the setting of increased NCX expression—as observed in heart failure or atrial fibrillation—NCX-mediated depolarization is enhanced leading to a prolongation of the action potential duration and favoring the occurrence of early and delayed afterdepolarizations (EADs, DADs). Thus, increased NCX function is regarded as a crucial factor for increased triggered activity and whole heart arrhythmia (Sipido et al., 2006; Pott et al., 2012; Bourgonje et al., 2013).

Conversely, we have recently shown that reduced NCX expression in a murine model with heterozygous knockout of the NCX gene leads to suppression of EADs and DADs (Bögeholz et al., 2015) supporting the above stated hypothesis. Others have evaluated the pharmacological (Nagy et al., 2004; Tanaka et al., 2007; Milberg et al., 2012) inhibition of NCX as an antiarrhythmic strategy. The majority of these studies used the NCX inhibitor SEA0400. Within the group of potent NCX inhibitors, previously represented by the peptide XIP (Li et al., 1991) and the isothiourea derivative KB-R7943 (Iwamoto et al., 1996), SEA0400 was introduced around the millennium. SEA0400 was considered as a major advance, especially due to its improved selectivity and high potency as compared to the mentioned precursors (Nagy et al., 2004). In later studies, the selectivity of SEA0400 has been debated and relativized (Reuter et al., 2002; Birinyi et al., 2005, 2008) due to relevant inhibitory efficacy on the L-type Ca^2+^ channel on higher concentrations (Birinyi et al., 2005). Other than XIP, SEA0400 is able to permeate the cell membrane. Encouragingly, the majority of studies to investigate the effect of SEA0400 on arrhythmia reported positive results, i.e., antiarrhythmic efficacy (Nagy et al., 2004; Milberg et al., 2012) in absence of negative inotropic effects (Bourgonje et al., 2013) thus potentially qualifying the concept of NCX inhibition as a promising antiarrhythmic strategy. However, there is also data suggesting unbeneficial effects of SEA0400 in the form of increased proarrhythmia (Ozdemir et al., 2008). These studies investigated various animal models and species in the absence or presence of heart failure and partially applied cardiac glycosides, that reduce the NCX forward mode function (O'Neill et al., 1990), to provoke arrhythmia (Nagasawa et al., 2005; Namekata et al., 2009; Nagy et al., 2014). Thus, NCX function was either enhanced due to an increased NCX expression in heart failure models or already reduced by cardiac glycosides which reduce the NCX driving force by increasing intracellular Na^+^. To the best of our knowledge, there is no study that evaluated the antiarrhythmic and inotropic effects of NCX inhibition by SEA0400 in dependence of the NCX expression and function level. However, this aspect is crucial, since major cardiac diseases like heart failure and atrial fibrillation (Voigt et al., 2012) are accompanied by NCX upregulation, which critically affects cardiac Ca^2+^ cycling. On the contrary, there are cardiac disease entities that exhibit a reduced NCX function, like the postinfarction (Zhang et al., 1996; Quinn et al., 2003) and diabetic cardiomyopathy (Schaffer et al., 1997; Hattori et al., 2000; Zhao et al., 2014). Thus, we here used a murine model of increased (Adachi-Akahane et al., 1997; Pott et al., 2012) or decreased (Henderson et al., 2004; Bögeholz et al., 2015) NCX expression (and consequently function) and corresponding wild-type mice to evaluate the effects of SEA0400 on Ca^2+^ transient amplitudes as a proxy measure for inotropy and the occurrence of proarrhythmic Ca^2+^ releases in dependence of the NCX expression level. Since chronic reduction of NCX expression and function has been demonstrated to prevent arrhythmia (Bögeholz et al., 2015), another more fundamental question to be addressed in this study is, whether acute pharmacological NCX inhibition similarly mediates antiarrhythmic effects. And if so, whether this antiarrhythmic effect does apply exclusively to the setting of increased NCX expression and/or function vs. reduced NCX function, bearing a potential answer toward the question whether there is an optimum of NCX function in the face of inotropy and arrhythmia.

## Methods

### NCX transgenic mouse models

Generation of global homozygous NCX overexpressor (OE) (Adachi-Akahane et al., 1997) and heterozygous knockout (hetKO) (Jordan et al., 2010) mouse model has been described previously. The genetic background of OE mice is C57BL/6 (Pott et al., 2012), whereas hetKO mice are on a combined genetic background of C57BL/6 and CD-1 due to embryonic transfer (Bögeholz et al., 2016). Animals entering experimentation were <12.5 weeks of age, since up to this age there is no evidence for structural remodeling or heart failure in OE (Pott et al., 2012) or hetKO mice (Jordan et al., 2010; Bögeholz et al., 2015). All experiments were approved by the local animal welfare authorities (Landesamt für Natur, Umwelt, und Verbraucherschutz NRW, permission number: 8.84-02.05.20.11.098) and conform to the guidelines from Directive 2010/63/EU of the European Parliament on the protection of animals used for scientific purposes.

### Isolation of ventricular cardiomyocytes

Ventricular cardiomyocytes were isolated from OE, hetKO and respective wild-type mice (WT_OE_/WT_hetKO_) as previously described with minor alterations (Schulte et al., 2012; Bögeholz et al., 2015). Mice were sedated and euthanized by carbon dioxide inhalation. Hearts were explanted immediately after decease and perfused retrogradely for 1 min via the aorta with a perfusion solution containing in mM: NaCl 113, KCl 4.7, KH_2_PO_4_ 0.6, Na_2_HPO_4_ 0.6, MgSO_4_ 1.2, NaHCO_3_ 12, KHCO_3_ 10, taurine 30, HEPES 10, glucose 11.1, BDM 10, heparin 14.3 IE/ml, pH = 7.4. To isolate rod-shaped viable ventricular myocytes, the extracellular connective tissue was degraded by perfusion for 6 min ± 10 s on 37.1°C with the perfusion solution containing Collagenase type 2 (Worthington Biochemical Corporation) and Protease from streptomyces griseus type XIV (Sigma Aldrich).

### Ca^2+^ epifluorescence measurements

Technical and procedural aspects of Ca^2+^ epifluorescence measurement have been described in detail before (Pott et al., 2012; Bögeholz et al., 2015). Data acquisition was performed using a Ca^2+^ imaging system, composed of a DeltaRAM monochromator and two Type-710 photomultiplier (Photon Technology International, USA) mounted to an inverse microscope (Diaphot 200, Nikon, Japan). Cardiomyocytes were transferred to a custom-made recording chamber and field-stimulated by platinum wires (Type 223 Stimulator CS, Hugo Sachs Elektronik- Harvard Apparatus, Germany). Fluorescence signals were recorded by FeliX 1.42 software (Photon Technology International, USA). The following external solution (Tyrode's solution) has been applied; in mM: NaCl 140, KCl 5.8, KH_2_PO_4_ 0.5, Na_2_HPO_4_ 0.4 MgSO_4_ 0.9, HEPES 10, glucose 11.1, CaCl_2_ 2, pH = 7.3. Experiments were performed at room temperature. The NCX function was assessed by the decay of caffeine (10 mM) induced Ca^2+^ transients (Bassani et al., 1994; Bögeholz et al., 2015, 2016) using fluo-4-AM (excitation: 488 nm, emission: 522 nm). The ratiometric fluorescent Ca^2+^ indicator indo-1-AM was employed to record field-stimulated Ca^2+^ transients and proarrhythmic spontaneous Ca^2+^ activity (excitation: 344 nm, emission-ratio: 405/495 nm). The amplitude of field-stimulated Ca^2+^ transients served as a proxy measure for inotropy. The most chosen SEA0400 concentration is 1 μM (Farkas et al., 2008; Namekata et al., 2009; Bourgonje et al., 2013; Nagy et al., 2014). Since previous studies demonstrated a relevant inhibitory effect of SEA0400 on other molecular targets next to NCX, such as the L-type Ca^2+^ channel, at concentrations higher than 1 μM (Birinyi et al., 2005) we decided not to exceed this threshold. Since we could observe an effect of SEA0400 on Ca^2+^ transient dynamics in initial dose-response experiments in a range between 0.3 and 1 μM, we decided to apply both concentrations.

### Statistics

Statistical calculations were conducted with SigmaPlot 11.2 (Systat Software; Inc.). Numerical results are expressed as mean value ± standard error of the mean (SEM). Number of replicates are indicated as *n* = number of independently analyzed cardiomyocytes/number of mice used for cardiomyocyte isolation. Data were analyzed by ANOVA on ranks followed by Dunn's *post hoc* test or repeated measures ANOVA followed by Holm-Sidak *post hoc* test. Rates and proportions were quantified using the Fisher's exact test with R.

## Results

### Effects of SEA0400 on NCX-mediated Ca^2+^ removal

To test the inhibitory effect of SEA0400 on the NCX forward mode, caffeine application experiments were conducted as reported previously (Bassani et al., 1994; Bögeholz et al., 2015). Before caffeine was applied, cells were field-stimulated in absence or presence of SEA0400, respectively, with 1 Hz for 1 min followed by 10 s of rest. A faster caffeine-induced Ca^2+^ transient decay indicates a higher NCX function.

**Figure 1:**
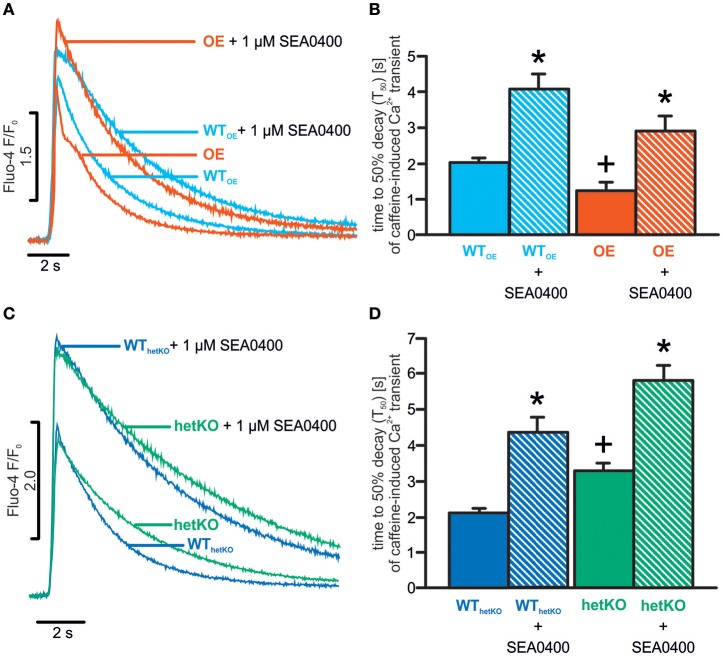
SEA0400 mediated NCX forward mode inhibition in caffeine application experiments. Representative tracings of caffeine-induced Ca^2+^ transients illustrate the different decay rates in OE **(A)**, hetKO **(C)** and respective WTs as a direct measure of NCX-mediated Ca^2+^ removal under basal conditions and during NCX inhibition by SEA0400. Quantification of time to 50% decay (T_50_) of caffeine-induced Ca^2+^ transients confirmed increased NCX forward mode function in OE **(B)** and decreased in hetKO **(D)**, and verified NCX forward mode inhibition by SEA0400 in all investigated groups but to different levels. ^*^p < 0.05 vs. basal; ^+^*p* < 0.05 OE/hetKO vs. WT_OE_/WT_hetKO_ basal; ANOVA on ranks test.

As demonstrated previously, in absence of SEA0400 (basal conditions), the time to 50% decay of caffeine-induced Ca^2+^ transients (T_50_) was significantly prolonged in heterozygous hetKO and shortened in OE as compared to the corresponding WT confirming a reduced/enhanced NCX-mediated Ca^2+^ removal rate, respectively (T_50_ in s: OE: 1.2 ± 0.2^+^; *n* = 37/5; WT_OE_: 2.0 ± 0.1; *n* = 42/5; hetKO: 3.3 ± 0.2^+^; *n* = 26/4; WT_hetKO_: 2.1 ± 0.2; *n* = 24/4; ^+^*p* < 0.05 OE vs. WT_OE_ or hetKO vs. WT_hetKO_; ANOVA on ranks test) (Figures 1A–D). When caffeine was applied in the presence of SEA0400 (1 μM), T_50_ was significantly increased vs. basal in all mouse lines indicating the inhibitory effect of SEA0400 on NCX forward mode (T_50_ in s; 1 μM SEA0400: OE: 2.9 ± 0.4^*^; *n* = 21/3; WT_OE_: 4.1 ± 0.4^*^; *n* = 25/3; hetKO: 5.8 ± 0.4^*^; *n* = 24/3; WT_hetKO_: 4.3 ± 0.4^*^; *n* = 24/3; ^*^p < 0.05 vs. basal; ANOVA on ranks test) (Figures 1B,D). In presence of SEA0400, T_50_ still tended to be shortened in OE and prolonged in hetKO compared to WT_OE_ and WT_hetKO_, respectively, however this finding was not statistically significant (*p* > 0.05 each; ANOVA on ranks test). Notably, the mean T_50_ value of OE in presence of SEA0400 was larger than in WT_OE_/WT_hetKO_ but still smaller than in hetKO under basal conditions indicating a remaining NCX function in OE ranging between the levels of WT_OE_/WT_hetKO_ and hetKO.

### Effects of SEA0400 on the Ca^2+^ transient amplitude

To test the effects of SEA0400 on the steady-state amplitude of field-stimulated Ca^2+^ transients, Ca^2+^ transients were measured in absence and presence of 0.3 and 1 μM SEA0400. Cells were field-stimulated at 1 Hz for 4 min followed by 20 sweeps on 0.125 Hz. This protocol was repeated in presence of 0.3 and 1 μM SEA0400 within the same cell. The amplitude of the Ca^2+^ transient was quantified at the end of the 1 Hz pacing phase after reaching a steady-state in Ca^2+^ transient kinetics. The Ca^2+^ transient amplitude in presence of SEA0400 is given in % of basal conditions.

**Figure 2:**
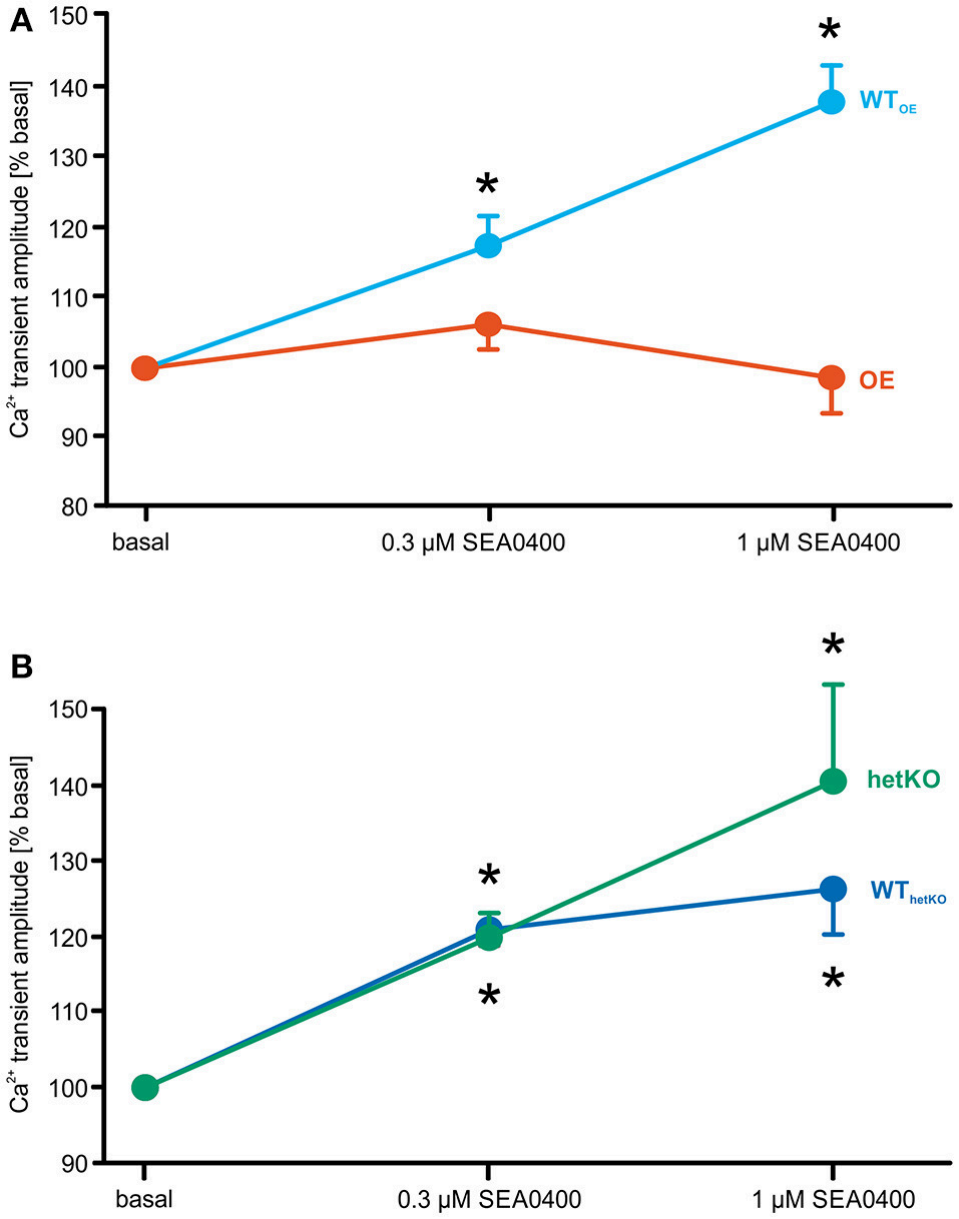
Relative effect of increasing SEA0400 concentrations on the amplitude of field stimulated Ca^2+^ transients as a measure for inotropy. SEA0400 mediated a significant relative increase of the Ca^2+^ transient amplitude vs. basal in WT_OE_ **(A)**, WT_hetKO_ and hetKO **(B)**, but not in OE **(A)**. Thus, the effect of SEA0400 on the Ca^2+^ transient amplitude seems to depend on the respective NCX expression level. ^*^p < 0.05 vs. basal; two-way repeated measures ANOVA test.

SEA0400 led to a similar relative increase of the mean Ca^2+^ transient amplitude in WT_OE_ (*n* = 27/7), WT_hetKO_ (*n* = 19/4), and hetKO (*n* = 19/5). However, in OE (*n* = 27/8) no increase of the Ca^2+^ transient amplitude was observed under application of SEA0400 (amplitude of field stimulated Ca^2+^ transients in % of basal; 1 μM SEA0400: OE: 98.7 ± 5.1; *n* = 23/8; WT_OE_: 137.8 ± 5.2^*^; *n* = 23/7; hetKO: 140.6 ± 12.8^*^; *n* = 9/3; WT_hetKO_: 126.3 ± 6.0^*^; *n* = 16/4; ^*^p < 0.05 vs. basal; repeated measures ANOVA test) (Figures 2A,B).

### Influence of SEA0400 on proarrhythmic spontaneous Ca^2+^ transients

To investigate the dependence of the antiarrhythmic effects of SEA0400 on NCX expression level, we monitored the occurrence of spontaneous Ca^2+^ releases (sCR), defined as spontaneously occurring Ca^2+^ transients between the field stimulated Ca^2+^ transients, during the 0.125 Hz pacing period in the above stated protocol before and after SEA0400 administration. An increase of sCR suggests an increase of proarrhythmia.

**Figure 3:**
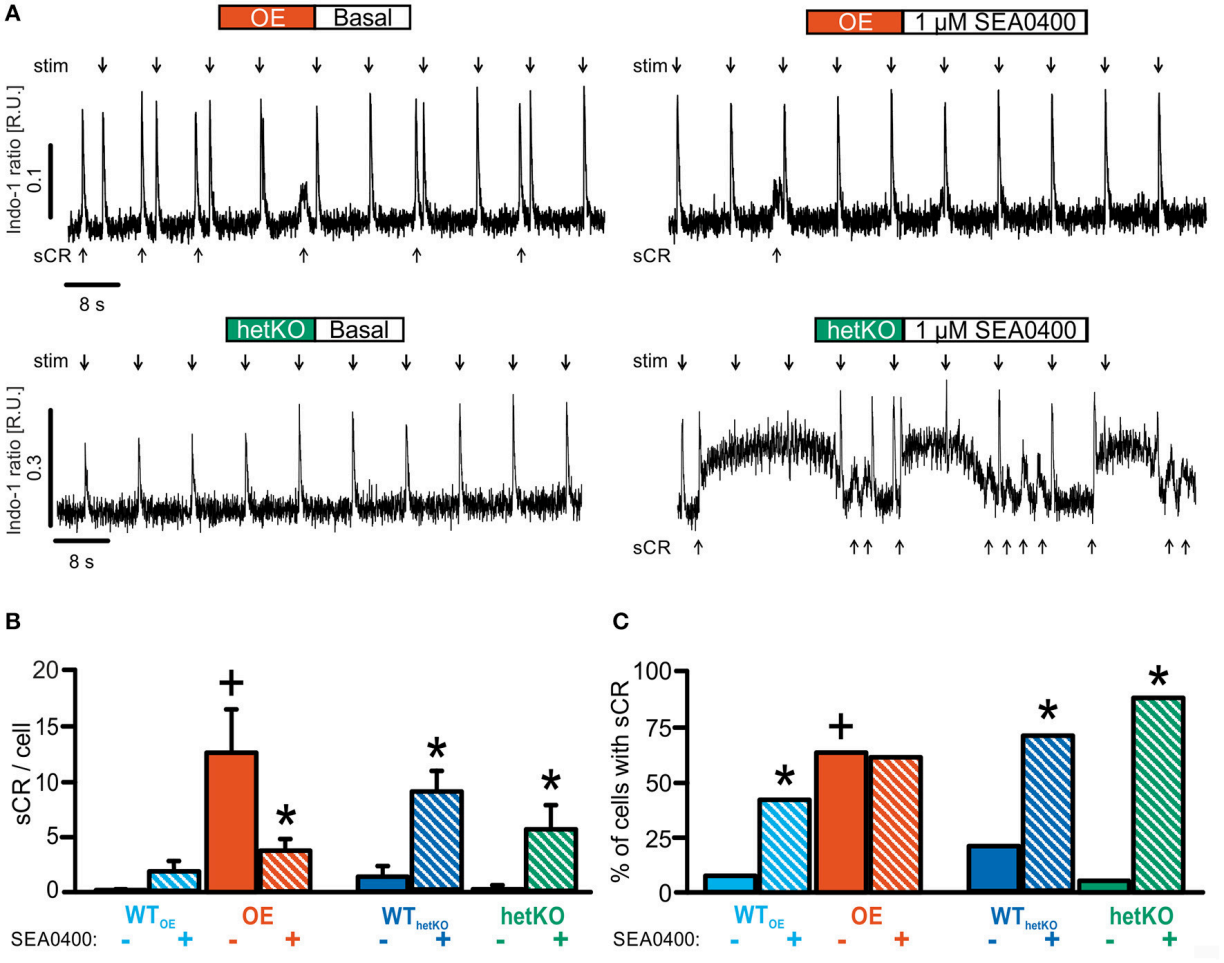
Quantification of spontaneous Ca^2+^ releases (sCR) as a measure for proarrhythmia. **(A)** Representative tracings illustrate the occurrence of numerous sCR (arrows below respective tracings) between field-stimulated Ca^2+^ transients (stim, arrows above respective tracings) in OE but not in hetKO in absence of SEA0400. **(B)** Quantification of sCR/cell under basal conditions and during exposure with 1 μM SEA0400. **(C)** Number of sCR-positive cardiomyocytes under basal conditions and during exposure with 1 μM SEA0400. SEA0400 significantly reduced the occurrence of sCR/cell in OE. Conversely, in hetKO SEA0400 increased the occurrence of sCR. Hence, the effect of SEA0400 on the occurrence of proarrhythmic spontaneous Ca^2+^ transients again depends on the NCX expression level. ^*^p < 0.05 vs. basal; ^+^*p* < 0.05 vs. corresponding WT basal; two-way repeated measures ANOVA test; *z*-test.

Under basal conditions, the occurrence of sCR (Figure 3) was similar to previously reported findings for OE (Pott et al., 2012) and hetKO (Bögeholz et al., 2015). The fraction of cells exhibiting spontaneous Ca^2+^ transients was significantly increased in OE compared to WT_OE_ (Figure 3C) (OE: 63%^+^, WT_OE_: 7%; ^+^*p* < 0.05 OE vs. WT_OE_, Fisher's exact test with R). The average number of sCR per cell (Figure 3B) was likewise increased in OE vs. WT_OE_ (number of sCR/all investigated cells: OE: 12.5 ± 3.8^+^; *n* = 27/8; WT_OE_: 0.2 ± 0.1; *n* = 27/7; ^+^*p* < 0.05 OE vs. WT_OE_; repeated measures ANOVA).

In hetKO the fraction of cells with sCR (Figure 3C) (hetKO: 5%; *n* = 19/5 WT_hetKO_: 21%; *n* = 19/4; *p* > 0.05 hetKO vs. WT_hetKO_, Fisher's exact test with R) as well as mean number of sCR per cell (Figure 3B) tended to be reduced vs. WT_hetKO_ but the results were not statistically significant (number of sCR/all investigated cells: hetKO: 0.2 ± 0.2; WT_hetKO_: 1.3 ± 1.0; *p* > 0.05 hetKO vs. WT_hetKO_; repeated measures ANOVA).

**Figure 4:**
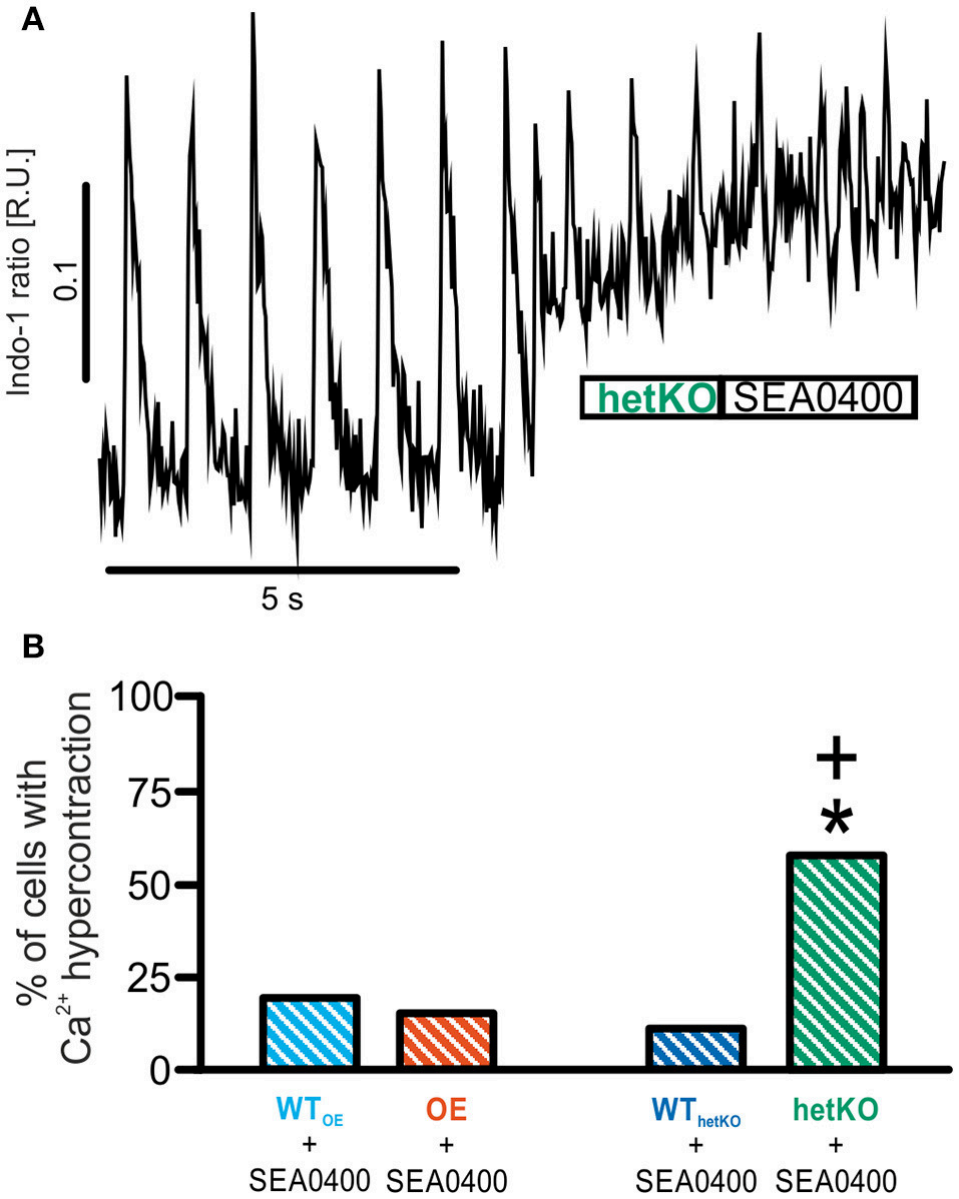
Quantification of Ca^2+^ hypercontractions as a measure for proarrhythmia. In a fraction of cardiomyocytes application of 1 μM SEA0400 caused a sudden increase of the diastolic Ca^2+^ level with highly frequent contractions and subsequent cell death (“Ca^2+^ hypercontraction”) **(B)** as illustrated by the representative tracing in **(A)**. The proportion of cells with Ca^2+^ hypercontractions was significantly increased only in the hetKO group. ^*^p < 0.05 vs. basal; ^+^*p* < 0.05 vs. WT; Fisher's exact test with R.

Application of SEA0400 altered the occurrence of sCR, but the specific effect depended upon the basal NCX expression level. In OE, SEA0400 significantly decreased the mean number of sCR per cell (Figure 3B) (number of sCR/all investigated cells; 1 μM SEA0400; OE: 3.7 ± 1.0^*^; *n* = 23/8; ^*^p < 0.05 vs. basal; repeated measures ANOVA) without altering the number of sCR-positive cells (Figure 3C). In contrast, we observed an increase in the occurrence of sCR in groups with normal or reduced NCX expression level. In WT_OE_ and WT_hetKO_ SEA0400 significantly increased the number of sCR-positive cells to 41 and 71% (Figure 3C). At the same time the occurrence of sCR per cell was increased significantly in WT_hetKO_ and in tendency in WT_OE_ in response to SEA0400 (Figure 3B) (number of sCR/all investigated cells; 1 μM SEA0400: WT_OE_: 2.4 ± 0.8; *n* = 23/7; WT_hetKO_: 8.8 ± 1.9^*^; *n* = 16/4; ^*^p < 0.05 vs. basal; repeated measures ANOVA). In hetKO application of 1 μM SEA0400 significantly increased both, the occurrence of sCR per cell (Figure 3B) (number of sCR/all investigated cells; 1 μM SEA0400: hetKO: 5.5 ± 2.2^*^; *n* = 8/3; ^*^p < 0.05 vs. basal; repeated measures ANOVA) and the number of sCR-positive cells (Figure 3C) (hetKO: 88%^*^; ^*^p < 0.05 vs. basal, Fisher's exact test with R). Moreover, SEA0400 drastically impaired Ca^2+^ homeostasis in hetKO as reflected by an abrupt elevation of the diastolic Ca^2+^ level, incessant Ca^2+^ oscillations and subsequent cell death, defined as Ca^2+^ hypercontraction (Figure 4), so that only 42% of all measured hetKO cells could be analyzed. The fraction of cells suffering from Ca^2+^ hypercontraction was significantly increased in hetKO as compared to WT_hetKO_ (Figure 4B) (hetKO: 58%^+^, WT_hetKO_: 11 %; ^+^*p* < 0.05 hetKO vs. WT_hetKO_, Fisher's exact test with R) but unaltered between OE and WT_OE_ (OE: 15%, WT_OE_: 19%; *p* > 0.05 OE vs. WT_OE_, Fisher's exact test with R).

## Discussion

In the search for novel strategies to counter cardiac arrhythmia, the cardiac Na^+^/Ca^2+^ exchanger (NCX) has been identified as a potential target. Due to its electrogenic mode of action (3 Na^+^: 1 Ca^2+^) NCX mediates membrane depolarization during Ca^2+^ extrusion. Hence, NCX may contribute to the development of EADs and facilitate the translation of spontaneous SR Ca^2+^ releases into DADs, which both may trigger arrhythmia (Sipido et al., 2006; Pott et al., 2012; Bourgonje et al., 2013). Consequently, pharmacological NCX inhibition should have antiarrhythmic effects but may on the other hand increase the cellular Ca^2+^ content, which could increase inotropy but also adversely promote arrhythmia. Encouragingly, initial work has demonstrated evidence for the antiarrhythmic efficacy of NCX inhibition mediated by SEA0400. However, the present study demonstrates, that the effects of SEA0400 on Ca^2+^ transient amplitude and proarrhythmia in murine cardiomyocytes are dependent on the NCX expression level.

### The effects of SEA0400 on the Ca^2+^ transient amplitude depend on the NCX function

SEA0400 reduced the NCX forward mode function to a similar extent in all mouse lines thus confirming inhibitory efficacy. At the same time, SEA0400 mediated a significant increase of the Ca^2+^ transient suggesting a positive inotropic effect in both wild-types (WT_OE_; WT_hetKO_) and hetKO but interestingly not in OE.

Previous studies found a positive inotropic effect in response to SEA0400 in murine, rat and pig models (Tanaka et al., 2005; Farkas et al., 2008; Ozdemir et al., 2008). Other studies reported rather neutral effects on contractility in rabbit, guinea pig and dog models (Nagy et al., 2004; Tanaka et al., 2007; Bourgonje et al., 2013). These diverse effects may be mostly attributable to differences in Ca^2+^ handling and basic electrophysiological properties between the various species. Analogous to cardiac glycosides, NCX inhibition can result in a net gain of intracellular Ca^2+^, which augments the Ca^2+^ transient amplitude and consequently inotropy. SEA0400 seems to mediate such a positive inotropic effect in smaller rodents (mouse and rat) but not in higher mammals, with the exception for pigs. In smaller rodents, the intracellular Na^+^ level is elevated compared to higher mammals which results in a reversal potential for NCX (E_Na/Ca_) close to the diastolic membrane potential (E_m_) reducing the driving force for the NCX forward mode (E_m_-E_Na/Ca_) (Bers, 2002). Moreover, Ca^2+^ removal from the cytosol in these species largely depends on sarco(endo-)plasmic reticulum Ca^2+^-ATPase (SERCA) which mediates the re-uptake of Ca^2+^ ions into the sarcoplasmic reticulum while NCX mediates Ca^2+^ extrusion to the extracellular compartment. In higher mammals, the competition between SERCA and NCX shifts in favor of NCX, although SERCA still removes the major proportion of cytosolic Ca^2+^ (Puglisi et al., 1996). To assure a balanced Ca^2+^ homeostasis, the exact amount of Ca^2+^ entering the cell mainly via L-type Ca^2+^ channels has to be extruded to the extracellular compartment via NCX. Thus, SEA0400-mediated reduction of a comparatively low NCX-mediated Ca^2+^ removal rate as observed in smaller rodents may more likely result in intracellular Ca^2+^ accumulation than in higher mammals which might explain the observed different inotropic responses in different studies. However, Ozdemir et al. found a positive inotropic response of SEA0400 in pigs as an example for higher mammals (Ozdemir et al., 2008). But they also found a lower NCX current in pig compared to mice, which might explain the positive inotropy via Ca^2+^ accumulation.

In contrast to both wild-types and hetKO, cardiomyocytes from OE mice did not exhibit a significant increase in the Ca^2+^ transient amplitude in response to SEA0400. In OE 1 μM SEA0400 reduced the NCX-mediated Ca^2+^ removal rate on average to a level ranging between the basal hetKO rate and the rate of both wild-types (Figures 1B,D). A NCX-mediated Ca^2+^ removal rate as low as in hetKO mice under basal conditions was not associated with a measurable increase of the amplitude of Ca^2+^ transients as reported previously (Bögeholz et al., 2015). Hence, the remaining NCX function in OE in presence of SEA0400 seems to be still high enough to cope with Ca^2+^ removal and thus to avoid net gain of Ca^2+^ and consequently an increase in Ca^2+^ transient amplitude. In this regard, the inhibitory effect of SEA0400 on L-type Ca^2+^ current has also to be taken into account (Birinyi et al., 2008). It has been shown that SEA0400 inhibits the L-type Ca^2+^ current either directly via pleiotropic effects, or indirectly by slowing the NCX-mediated Ca^2+^ removal leading to an increase of subsarcolemmal Ca^2+^ concentration which in turn promotes Ca^2+^ dependent inactivation of L-type Ca^2+^ current (Pott et al., 2005; Bögeholz et al., 2015).

Interestingly, Ozdemir et al. similarly found absence of a positive inotropic effect in MLP-/- mice, a murine model of heart failure (Ozdemir et al., 2008). Due to the heart failing phenotype NCX upregulation is likely, which may prevent from Ca^2+^ accumulation and positive inotropy. Taken together within the same species the effect of the same concentration of SEA0400 on calcitropy and thus inotropy seems to depend on the NCX expression level and corresponding function.

### Effects of SEA0400 on cellular proarrhythmia depend on the NCX expression level

In previous studies, SEA0400 exhibited antiarrhythmic efficacy on cellular level by suppressing afterdepolarizations (Nagy et al., 2004; Zhao et al., 2012), on whole heart level *in vitro* (Milberg et al., 2008, 2012) and even *in vivo* in a canine model (Nagasawa et al., 2005; Bourgonje et al., 2013). On the other hand, proarrhythmic effects mediated by SEA0400 have been reported as well (Ozdemir et al., 2008). The respective NCX function might be considerably heterogeneous in the investigated animal models, i.e., increased in the case of heart failure, or effectively decreased in the setting of cardiac glycoside-induced proarrhythmia. We here demonstrate that the effects of SEA0400 on cellular proarrhythmia again depend on the underlying baseline NCX function within the same species. Considering NCX inhibition as a promising therapeutic strategy, this might be of translational relevance, since major cardiac diseases like heart failure or atrial fibrillation are accompanied by NCX upregulation, whereas other specific cardiac disease entities, like the postinfarction (Zhang et al., 1996; Quinn et al., 2003) and diabetic cardiomyopathy (Schaffer et al., 1997; Hattori et al., 2000) exhibit a reduced NCX function, at least in animal models. There also might be further specific disease entities with altered NCX function that are yet to be further evaluated (Primessnig et al., 2016).

### SEA0400 reduced cellular proarrhythmia in OE

In OE, application of SEA0400 reduced the occurrence of spontaneous Ca^2+^ transients. In a former study the increased NCX expression and function in OE has been identified as an independent proarrhythmic factor (Pott et al., 2012). Thus, the observed reduction of arrhythmogenic spontaneous Ca^2+^ transients in OE can be attributed to the SEA0400-mediated inhibition of NCX function.

The underlying molecular mechanism of SEA0400-mediated decrease in the occurrence of spontaneous Ca^2+^ transients in OE may consist of reduced NCX forward mode with subsequently reduced depolarizing inward current thus avoiding membrane depolarization and the occurrence of EADs and DADs as proarrhythmic triggers. However, in this regard again the inhibitory potency of SEA0400 on L-type Ca^2+^ current has to be taken into account (Birinyi et al., 2008; Bourgonje et al., 2013), which might contribute to the observed reduction of proarrhythmic spontaneous Ca^2+^ transients in OE in presence of SEA0400. Supplementary to these molecular mechanisms of arrhythmia, we have previously demonstrated a significant antiarrhythmic effect of SEA0400 on whole-heart arrhythmia (ventricular tachycardia) in a rabbit model of heart failure (Milberg et al., 2012). SEA0400 reduced the occurrence of EADs due to a reduced monophasic action potential duration, dispersion and QT interval. Thus, the antiarrhythmic effects of SEA0400 may be diverse and apply to diverse functional levels.

### Proarrhythmic effects of SEA0400 in wild-type and hetKO

In contrast to OE, SEA0400 led to an increased occurrence of spontaneous Ca^2+^ transients in WT_OE_, WT_hetKO_, and hetKO. Furthermore, Ca^2+^ homeostasis seemed to be drastically impaired in hetKO treated with SEA0400 as reflected in the increase percentage of cardiomyocytes with hypercontraction and subsequent cell death as a consequence of cytosolic Ca^2+^ overload. Based on these observations, it seems likely that a critical level of NCX function must remain after SEA0400 treatment to avoid detrimental intracellular Ca^2+^ accumulation and overload. While the reduced basal NCX function in hetKO led to a phenotype with reduced likelihood of arrhythmia and a balanced Ca^2+^ homeostasis as reported previously (Bögeholz et al., 2015), lowering NCX function below this level with SEA0400 was clearly detrimental in hetKO, WT_hetKO_, and WT_OE_. In this regard, one has to distinguish between acute reduction of NCX function via SEA0400 and chronically reduced NCX function via transgenic manipulation. The latter offers the opportunity for long-term adaption beginning from the early stages of development to compensate for the reduced Ca^2+^ removal rate mediated via NCX. The most important adaptive mechanism in models with reduced (Bögeholz et al., 2015) or absent NCX function (Pott et al., 2007a,b,c) to keep a balanced Ca^2+^ homeostasis consists of a reduced Ca^2+^ entry via the L-type Ca^2+^ channel as mediated by an enhanced Ca^2+^-dependent inactivation of the L-type Ca^2+^ current. Thus, if less Ca^2+^ enters the cell, the reduced NCX function is still sufficient to keep a balanced Ca^2+^ homeostasis in a chronically adapted system. In contrast, the lack of time for adaptation to acute NCX inhibition by SEA0400 may contribute to the impaired Ca^2+^ homeostasis in WT and most prominently in hetKO. However, in OE acute application of SEA0400 was not detrimental but reduced the cellular proarrhythmia through acute NCX inhibition. Thus, depending on the baseline NCX expression level, acute SEA0400 application can cause destabilization of intracellular Ca^2+^ homeostasis leading to arrhythmia and cell death or mediate antiarrhythmic effects.

## Conclusion and translational perspective

In conclusion, our results suggest that within the same species beneficial or detrimental effects mediated by SEA0400 depend upon the basal NCX expression and thus function level. A reduced NCX function level as low as that in hetKO has been shown to be antiarrhythmic (Bögeholz et al., 2015). Reducing an already increased proarrhythmic NCX function level, such as that in OE (Pott et al., 2012), by SEA0400 to the baseline hetKO level reduces proarrhythmia without increasing the amplitude of Ca^2+^ transients. Reducing the NCX function level below this value increases the amplitude of Ca^2+^ transients due to SR Ca^2+^ loading but at the same time mediates cellular proarrhythmia as demonstrated in WT_hetKO_, WT_OE_ and most prominently in hetKO where the remaining NCX function is the lowest. Consistent with this observation, a previous study demonstrated an enhanced susceptibility toward arrhythmia in a ventricular specific homozygous NCX knockout mouse model with absence of NCX expression in 80–90% of ventricular cardiomyocytes (Jordan et al., 2010). Taken together, these findings suggest that NCX inhibition may be beneficial, however there seems to be a critical level of the remaining NCX function that is needed for balanced Ca^2+^ homeostasis. In case of falling below a certain critical threshold, adverse effects occur due to Ca^2+^ accumulation promoting proarrhythmia (Table 1). Translated into the clinical context, NCX inhibition may be antiarrhythmic in absence of a negative inotropic effect in conditions of increased NCX expression as present in heart failure or atrial fibrillation. Albeit, in conditions of preexisting reduced NCX function, as observed in animal models of postinfarction or diabetic cardiomyopathy, additional NCX inhibition may potentially exert proarrhythmic effects accompanied by increased inotropy due to a reduction of the remaining NCX function below a critical level that is needed for balancing the cellular Ca^2+^ dynamics. Thus, at-risk groups should be identified in future large-scaled clinical trials investigating specific NCX inhibitors.

**Table 1:** Summary of the effects of SEA0400 on Ca^2+^ handling in dependence on the NCX expression level.

**NCX expression**	**OE**	**WT_OE_**	**WT_hetKO_**	**hetKO**
	**320%**	**100%**	**100%**	**47%**
	**SEA0400 effect**			
Remaining NCX function	↓	↓↓	↓↓	↓↓↓
Ca^2+^ transient amplitude	↔	↑	↑	↑
Proarrhythmic effect	↓	↑	↑	↑↑
Antiarrhythmic effect	↑	↓	↓	↓↓

*SEA0400 reduced the NCX forward mode function in all mouse lines but to different remaining NCX function levels. This results in a positive inotropic effect in the presence of unaltered or reduced NCX expression in hetKO and both WTs, but not in face of increased NCX expression in OE. SEA0400 reduced spontaneous Ca^2+^ transients in OE but oppositely mediated a proarrhythmic effect under conditions of unaltered or reduced NCX expression. These comparative results indicate that the occurrence of detrimental or beneficial effects of SEA0400 depend on the remaining NCX function present during NCX inhibition*.

### Limitations

In this study, the Ca^2+^ transient amplitude served as a proxy measure for inotropy on the cellular level. Though an acute increase of the Ca^2+^ transient amplitude is usually accompanied by a simultaneous increase in contractility, we did not measure changes in contractility directly. Likewise, although spontaneous Ca^2+^ releases represent a well-known proarrhythmic substrate, it has to be acknowledged, that clinically relevant whole-heart arrhythmia is a more complex result from a magnitude of involved factors. Thus, future studies should investigate the *in vivo*-relevance of our findings derived from the cellular level.

Moreover, our findings may not be uncritically transferable into the human (patho-)physiology, since there are substantial differences in cardiac Ca^2+^ cycling und electrophysiology compared to a murine model. It remains also to be tested whether more specific NCX inhibitors show comparable effects to SEA0400. However, despite these limitations our data suggest that care should be taken when testing NCX inhibitors in clinical studies where we usually do not know the respective NCX expression and function level of individuals, which might determine beneficial or detrimental effects of the treatment approach with an NCX inhibitor.

## Author contributions

NB, JS, SK, CP, and FM designed the study. NB, JS, SK, BB, and PP acquired the data. NB, JS, SK, BB, and DD performed the statistical analysis. All authors contributed substantially to the interpretation of the data. NB, JS, and SK wrote the manuscript. BB, PP, DD, GF, JG, UK, LE, CP, and FM critically revised the manuscript. All authors approved the final version of the manuscript.

### Conflict of interest statement

The authors declare that the research was conducted in the absence of any commercial or financial relationships that could be construed as a potential conflict of interest.
